# Association of post-operative ctDNA detection with outcomes of patients with early breast cancers

**DOI:** 10.1016/j.esmoop.2024.103687

**Published:** 2024-08-30

**Authors:** R. Cutts, L. Ulrich, M. Beaney, M. Robert, M. Coakley, C. Bunce, G.W. Crestani, S. Hrebien, E. Kalashnikova, H.-T. Wu, S. Dashner, H. Sethi, A. Aleshin, M. Liu, A. Ring, A. Okines, I.E. Smith, P. Barry, N.C. Turner, I. Garcia-Murillas

**Affiliations:** 1 The Breast Cancer Now Toby Robins Research Centre, The Institute of Cancer Research, London; 2 Breast Unit, Royal Marsden Hospital, London, UK; 3 Department of Breast Medical Oncology and Neuro-Oncology, Early Therapeutic Unit, Institute of Oncology de l'Ouest, St Herblain, France; 4 Clinical Trials Unit, Royal Marsden Hospital, London, UK; 5 Natera, Inc., Austin, USA; 6 The Ralph Lauren Centre for Breast Cancer Research, Royal Marsden Hospital, London, UK

**Keywords:** breast cancer, ctDNA, molecular residual disease

## Abstract

**Background:**

In early breast cancer (EBC) patients, we aimed to determine whether circulating tumor DNA (ctDNA) analysis following primary surgery, before systemic therapy, identified molecular residual disease and was associated with risk of relapse and relapse-free survival (RFS).

**Methods:**

Plasma was collected, retrospectively, before surgery, 1-14 weeks post-operatively, and before adjuvant therapy, and in a subset of patients after adjuvant therapy. A personalized, tumor-informed, multiplex PCR next generation sequencing assay (Signatera™) was used for ctDNA detection and quantification. The primary objective was to compare RFS and distant recurrence-free survival (DRFS) in patients with detected versus non-detected ctDNA.

**Results:**

A total of 48 patients with EBC (median age 50.5 years) [34 hormone receptor-positive/human epidermal growth factor receptor 2-negative (HR+/HER2−), 5 HER2+, 9 triple-negative breast cancer) were included. ctDNA was detected in 64.5% (20/31) of patients before surgery, and 35.4% (17/48) after surgery. ctDNA detection before surgery was associated with tumor grade (*P* = 0.019), ctDNA detection after surgery was associated with receptor subtype (*P* = 0.01). Patients with ctDNA detected after surgery had worse DRFS [hazard ratio = 5.5, 95% confidence interval (CI) 1.1-28.5, *P* = 0.04]. RFS in patients with ctDNA detected after surgery was worse than in those with lack of ctDNA detection, although not statistically significant (hazard ratio = 3.7, 95% CI 0.9-15.7, *P* = 0.073). Patients with ctDNA detected preoperatively or post-operatively had a trend towards worse RFS (hazard ratio = 7.8, 95% CI 0.9-63.7, *P* = 0.05) and DRFS (hazard ratio = 6.8, 95% CI 0.8-57, *P* = 0.07) compared with those with ctDNA undetected at both timepoints. ctDNA detection anticipated clinical relapse with a median lead time of 16 months.

**Conclusions:**

In patients with treatment-naive EBC, ctDNA is detectable after surgery. The absence of ctDNA at a single post-surgical timepoint is associated with improved DRFS, supporting the development of future trials studying de-escalation of systemic therapy.

## Highlights

• In systemic treatment-naive early breast cancer patients, ctDNA is detectable after surgery.
• Presence of ctDNA at a single post-surgical timepoint is associated with distant metastasis-free survival.
• Detection of ctDNA post-operatively might offer a route to identify patients who could benefit from adjuvant chemotherapy.

## Introduction

In women, breast cancer is the most commonly diagnosed cancer globally and the second most frequent cause of cancer-related death.^1^ The majority of patients with breast cancer present with early-stage, primary breast cancer with no evidence of overt metastatic disease.^2^ Although advances in diagnosis and treatment have led to substantial improvement in disease-free survival over the past few decades, there is still a need for better tools for post-local therapy risk stratification. Ideal tools would inform adjuvant therapy selection for tailored treatment such that those at highest risk of relapse would receive the most effective adjuvant therapies and those at low risk could be spared the toxicities of additional therapies.

Presently, the surveillance of breast cancer following definitive therapy includes routine physical examination and breast imaging as appropriate. Circulating tumor DNA (ctDNA) has emerged as a non-invasive tool to detect molecular residual disease (MRD) with potential to guide adjuvant therapy administration and recurrence monitoring. In solid tumors, MRD detection by ctDNA has been reported in breast,^3^, ^4^, ^5^, ^6^, ^7^ colon,^8^, ^9^, ^10^, ^11^ and lung^12^^,^^13^ among other cancers. Most of these studies have relied on serial sampling and identification of ctDNA along the follow-up period rather than identification of MRD immediately following surgery or during the early adjuvant setting. Recently presented data in colon cancer^14^ suggest that identification of patients with detectable ctDNA following primary treatment can identify those at high risk of disease progression, and therefore may impact frequency of surveillance and/or inform decisions for the administration of guided adjuvant therapies. Finally, post-operative ctDNA analysis has the potential to identify patients at high risk for treatment intensification as well as informing the future design and development of clinical trials of novel adjuvant therapies.

Prior research on ctDNA-detected MRD has focused on patients who have received both chemotherapy and surgery. Data specifically on ctDNA detection after primary surgery and before adjuvant therapy would enable assessment of ctDNA-guided adjuvant therapy decisions. In this study, we carried out longitudinal ctDNA assessment using a personalized, tumor-informed multiplex PCR (mPCR) next generation sequencing (NGS) assay (Signatera™, San Carlos, CA) to identify MRD after surgery. We evaluated the association of post-operative ctDNA detection with relapse-free survival (RFS) and distant recurrence-free survival (DRFS) and provide evidence that post-operative MRD detection in this setting is associated with worse outcomes.

## Methods

### Patient cohort

For this retrospective, proof of-principle study, patients with early breast cancer (EBC), irrespective of hormone receptor (HR)/human epidermal growth factor receptor 2 (HER2) status, undergoing primary surgery for breast cancer (*N* = 48), enrolled in the PlasmaDNA (REC Ref No: 10/H0805/50) and ITH (REC Ref No: 13/LO/1015) sample collection studies, were included in this analysis (Supplementary Figure S1, available at https://doi.org/10.1016/j.esmoop.2024.103683). Both studies were approved by the East of England Health Research Authority ethics committee. Written informed consent was obtained from all participants. All patients had primary breast cancer without evidence of distant metastatic disease, with staging scans conducted according to local guidelines. After completion of surgery patients were treated with adjuvant hormone therapy, chemotherapy, or trastuzumab as per standard local practice. Four patients had neoadjuvant chemotherapy before any blood sample collection. All patients were recruited to this study based on available plasma samples collected after surgery and before any adjuvant therapy initiation. Clinical and histopathological information was collected for all patients (Supplementary Tables S1 and S2, available at https://doi.org/10.1016/j.esmoop.2024.103683).

### Sample collection

Formalin-fixed paraffin embedded (FFPE) tumor tissue was collected at the time of surgery or at the time of initial diagnosis following standard procedures. Whole peripheral blood for plasma separation was retrospectively collected using EDTA tubes and processed within 2 h following venipuncture or Streck tubes and processed within 24-48 h following venipuncture and before surgery (*n* = 31), 1-14 weeks post-operatively (*n* = 48) before commencing any adjuvant therapy, and during follow-up (*n* = 17) after completion of adjuvant chemotherapy. ctDNA extraction and detection underwent batched retrospective analysis by Natera, Inc., San Carlos, CA blinded to patient outcomes and sample order. Neither patients nor providers received the Signatera results from this study.

### Whole exome sequencing of tumor and germline DNA to identify mutations for tracking

Tumor DNA from FFPE tissue and normal DNA from the buffy coat of whole blood was whole exome sequenced (WES) using either the IDT xGen Exome Hyb Panel v1 or Agilent SureSelect V6 to a median deduplicated depth of 290× (range 141× to 1474×) in tumor DNA and 112× (range 15× to 338×) in germline DNA to identify tumor-specific somatic single nucleotide variants (SNVs). Briefly, fastq files were aligned to the human genome version GRChr37 using bwa v0.7.12.^15^ SNVs were identified using three mutation callers, GATK Mutect2 v4.1.3.0,^16^ VarScan 2,^17^ and VarDict.^18^ GATK Picard metrics^16^ was used to assess sequencing quality and sample purity was assessed using TitanCNA.^19^ SNVs were ranked and personalized mPCR-based NGS assays were designed.

### Personalized mPCR-based NGS assay for ctDNA detection

A personalized, tumor-informed, mPCR NGS assay (Signatera™) was used for the detection and quantification of ctDNA. Briefly, all patients had 16 SNVs identified by WES, except one patient who had 12 variants identified. These were selected for each patient specific panel. mPCR primer pairs were designed and synthesized for the selected SNVs to track ctDNA in plasma samples. Cell-free DNA was extracted from a median of 3.6 ml of plasma (range 1.8-4.7 ml). Universal libraries were synthesized by end repair, A-tailing, and ligation with custom adapters. Next, libraries were amplified by mPCR, barcoded, pooled, and sequenced on an Illumina NGS platform. Plasma samples with at least two variants detected were defined as ctDNA detected. ctDNA concentration was reported in mean tumor molecules (MTM)/ml of plasma.^5^

### Statistical analysis

The primary objective of this study was to assess the association between post-operative ctDNA detection with RFS and DRFS. Secondary objectives were (i) to assess whether ctDNA detection at a pre-surgical timepoint was associated with RFS and DRFS and (ii) to compare RFS and DRFS between patients with ctDNA detection either before or after surgery and patients with no detectable ctDNA at these timepoints. Univariable analysis using log-rank test and cox proportional hazard model with 95% confidence intervals (CIs) were used. Univariable and multivariable variables included histological subtype, receptor status, tumor grade and nodal involvement. Patients who developed second primary malignancies were censored. All statistical analysis was conducted using Stata v17, with statistical significance set at the 5% level and all *P* values were two-sided.

## Results

A total of 115 plasma samples were collected from 48 patients with invasive ductal carcinoma (*n* = 39), invasive lobular carcinoma (*n* = 8), and metaplastic breast cancer (*n* = 1). Clinicopathological characteristics were collected for all patients (Supplementary Tables S1 and S2, available at https://doi.org/10.1016/j.esmoop.2024.103683). The median age of the cohort was 50.5 years (range 28-87 years). Median follow-up time from surgery was 60 months (range 24-72 months).

### Presurgical ctDNA detection

**Figure 1:**
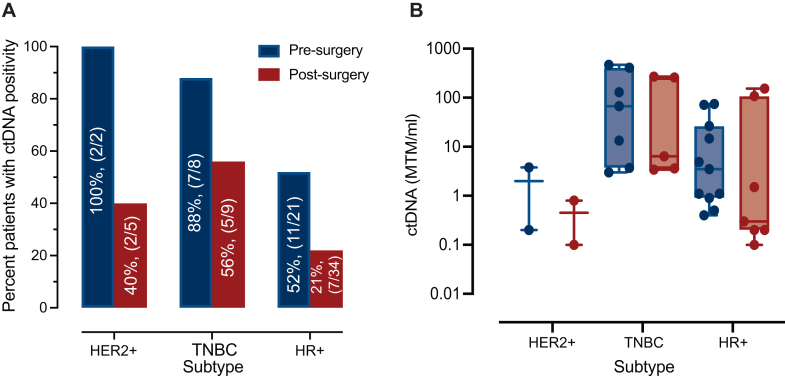
**ctDNA detection rates before and after surgery.** (A) ctDNA detection rates before and after surgery by subtype, (B) ctDNA levels before and after surgery by subtype. ctDNA, circulating tumor DNA; HER2, human epidermal growth factor receptor 2; HR, hormone receptor; MTM, mean tumor molecules; TNBC, triple-negative breast cancer.

ctDNA was detected at a single pre-surgery timepoint in 20/31 patients (65%). Higher detection rates were observed in cases with HER2+ (100%, 2/2) and triple-negative breast cancer (TNBC) (88%, 7/8) disease, followed by HR+/HER2− (52%, 11/21) positive subtype (Figure 1A).

Highest median MTM/ml levels were observed in TNBC [67.3 (range 3-469.9)] followed by HER2+ [2 (range 0.2-3.8)] and HR+/HER2− [3.5 (range 0.4-74.2)] patients (Figure 1B). Of the four patients who received neoadjuvant treatment three remained ctDNA detectable when tested before surgery.

Univariable analysis revealed that ctDNA detection before surgery was associated with tumor grade (*P* = 0.019; Fisher’s exact test). We found no association between ctDNA detection and histology, subtype, or nodal involvement, although these analyses were limited by the small sample size. In multivariable analyses, ctDNA detection before surgery was associated with subtype (*P* = 0.01), with more aggressive subtypes like TNBC having a larger proportion of ctDNA detected patients at this timepoint, 88% (7/8) compared with HR+/HER2− patients (52%, 11/21).

### Post-operative ctDNA status and survival

ctDNA analysis was carried out on post-operative plasma samples collected before initiation of adjuvant therapy. ctDNA detection rates for all subtypes decreased after surgery [HER2+: 40% (2/5), TNBC: 56% (5/9), and HR+: 21% (7/34)] (Figure 1A). Similarly, ctDNA concentration was observed to be lower after surgery [tested at a median of 0.5 months (0.2-2.4 months)], compared with the pre-surgery levels. Median ctDNA levels were as follows: HER2+ [0.45 MTM/ml (0.1-0 MTM/ml)], TNBC [6.4 MTM/ml (3.4-268.6 MTM/ml)], and HR+/HER2− disease [0.3 MTM/ml (0.1-154 MTM/ml)] (Figure 1B). No statistically significant association of ctDNA detection with histology, tumor grade or nodal involvement was observed in this cohort at the post-surgery timepoint. The positive predictive value (PPV) for relapse detection at a single post-surgery timepoint was 35.7% (95% CI 20.2% to 55%) while the negative predictive value (NPV) was 91.2% (95% CI 80.6% to 96.2%).

**Figure 2:**
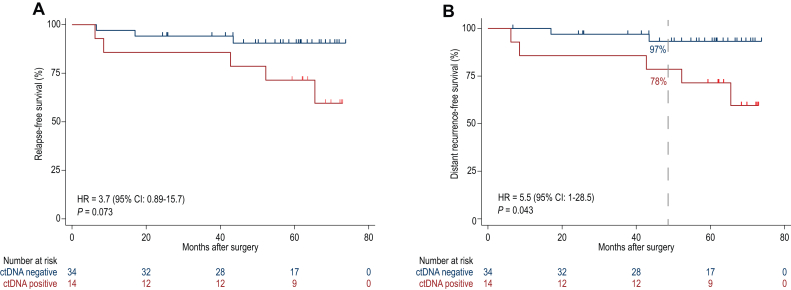
**ctDNA detection associates with worse RFS and DRFS.** (A) Patients with ctDNA detected at a single post-surgery timepoint had worse RFS and (B) DRFS (log-rank test). Dashed line shows the DRFS landmark and survival proportions. CI, confidence interval; ctDNA, circulating tumor DNA; DRFS, distant recurrence-free survival; HR, hazard ratio; RFS, relapse-free survival.

RFS in patients with ctDNA detected after surgery was worse than in those with lack of ctDNA detection, although not statistically significant (hazard ratio = 3.7, 95% CI 0.9-15.7, *P* = 0.073) (Figure 2A). ctDNA detection was associated with worse DRFS with statistical significance (hazard ratio = 5.5, 95% CI 1-28.5, *P* = 0.043) (Figure 2B). We observed a 78% (95% CI 47% to 92%) DRFS at 4-years follow-up in those patients with ctDNA detected at this timepoint compared with 97% (95% CI 80% to 99%) in those without ctDNA detection after surgery.

**Figure 3:**
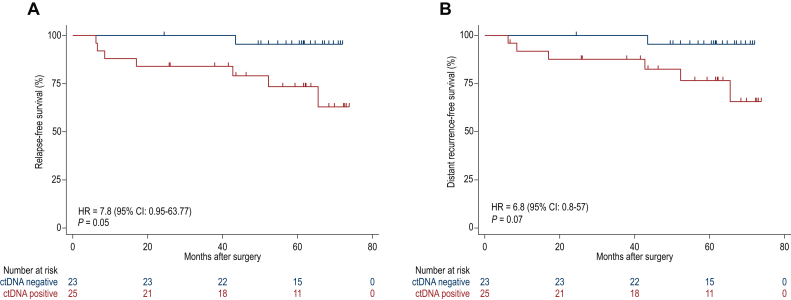
**ctDNA detection pre- or postoperatively associates with worse RFS and DRFS.** (A) Patients with ctDNA detected either pre- or postoperatively had worse RFS and (B) DRFS (log-rank test). CI, confidence interval; ctDNA, circulating tumor DNA; DRFS, distant recurrence-free survival; HR, hazard ratio; RFS, relapse-free survival.

We explored the association of combined ctDNA status before and after surgery (before adjuvant therapy) with risk of recurrence. We observed that those with ctDNA detected at either of these timepoints had worse RFS (hazard ratio = 7.8, 95% CI 0.95-63.77, *P* = 0.05) (Figure 3A) and DRFS (hazard ratio = 6.8, 95% CI 0.8-57, *P* = 0.07) (Figure 3B) compared with patients who were ctDNA undetected at both timepoints.

### ctDNA status during follow-up and recurrence

**Figure 4:**
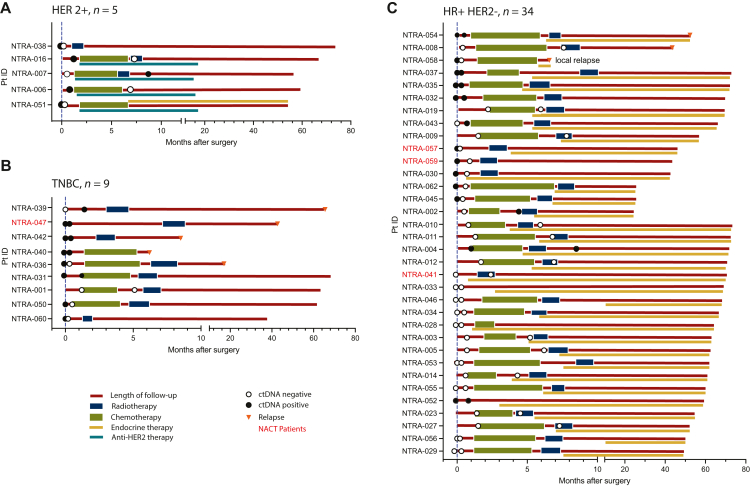
**Patient overview plot indicating association of breast cancer subtype with disease recurrence and survival.** (A) HER2+ patients, (B) TNBC patients, and (C) HR+ HER2− patients. Black circles (ctDNA detected), white circles (ctDNA undetected), and red triangles relapse. Olive bars indicate adjuvant chemotherapy duration, blue bars indicate radiotherapy, yellow bars indicate endocrine, teal bases represent anti-HER2+ treatment, and red bars indicate follow-up. Patients IDs who received neoadjuvant chemotherapy are highlighted in red. ctDNA, circulating tumor DNA; HER2, human epidermal growth factor receptor 2; HR, hormone receptor; NACT, neoadjuvant chemotherapy; TNBC, triple-negative breast cancer.

Some 35% (17/48) of patients had a follow-up blood sample collected after completion of adjuvant chemotherapy. Of these, 76% (13/17) had HR+ disease, and all but 1 patient received adjuvant endocrine therapy with or without radiotherapy. Three patients had HER2+ disease and were undergoing anti-HER2+ treatment (with or without radiotherapy), one patient had triple-negative disease. Of these 17 patients, 4 (24%) had detectable ctDNA. None of these patients experienced disease progression: three patients with HR+ disease continued to receive adjuvant endocrine therapy (with or without radiotherapy) after ctDNA detection and one patient (HER2+) was undergoing anti-HER2+ treatment (Figure 4). ctDNA detection anticipated clinical relapse with a median lead time of 16 months.

Among the 13 patients without ctDNA detection during follow-up, 1 patient experienced recurrence after completion of adjuvant chemotherapy. This patient had HR+ disease but did not receive endocrine therapy and experienced a relapse ∼43 months after the last blood sample was collected. Notably, the remaining 12 patients in this group remained recurrence free for a median follow-up of 56 months (range 44.8-65.1 months) after the last blood test (Figure 4).

### ctDNA status after surgery and response to chemotherapy

**Figure 5:**
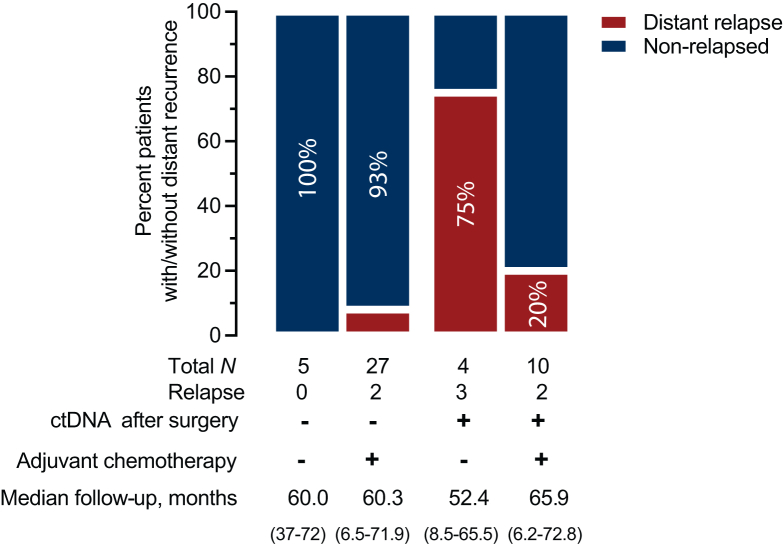
**Presence of ctDNA after surgery might help identify those patients who respond to adjuvant chemotherapy.** ctDNA, circulating tumor DNA.

Our results indicate that the presence of ctDNA after surgery might be associated with a likelihood of response to adjuvant chemotherapy. Interestingly, out of 34 of patients with ctDNA undetected, only 7% (2/29) of those who received adjuvant chemotherapy experienced DRFS (Figure 5), and no events were observed among 5 patients who did not receive adjuvant chemotherapy. Patients with detected ctDNA (*n* = 14) experienced a higher rate of relapse—without adjuvant chemotherapy: 75% (3/4) versus with adjuvant chemotherapy: 20% (2/10) (Figure 5).

With limited cohort size, however, this observation suggests that adjuvant chemotherapy efficacy may be different for those with detectable post-operative MRD versus those without detectable MRD (Supplementary Figure S2A, available at https://doi.org/10.1016/j.esmoop.2024.103683). In contrast, patients with lack of ctDNA detection after surgery demonstrated more favorable outcomes in our study; no additional benefit of receiving chemotherapy was observed for this group of patients (Supplementary Figure S2B, available at https://doi.org/10.1016/j.esmoop.2024.103683).

## Discussion

There is currently a need to identify biomarkers capable of detecting MRD after surgery and predicting disease recurrence following operative management to improve decisions regarding adjuvant therapy in patients diagnosed with early-stage breast cancer. In this cohort of EBC patients undergoing primary surgery, we utilized ctDNA analysis to identify MRD following primary resection and found that post-surgical ctDNA detection is associated with future relapse. In this study we mostly identified early recurrences, as most of the cases were HR+/HER2−, in which more than half of the recurrences occur beyond the median follow-up of this study. Some 97% (26/27) of patients who were ctDNA-undetected after surgery and before adjuvant therapy remained without distant relapse at 4 years of follow-up.

In evaluating the association of ctDNA detection with survival, we found that RFS (hazard ratio = 3.7, 95% CI 0.9-15.6, *P* = 0.07) and DRFS (hazard ratio = 5.5, 95% CI 1.1-28.5, *P* = 0.04) at a single post-surgical timepoint were worse in those patients who were ctDNA-detected compared with ctDNA-undetected patients. Although the association with RFS was not statistically significant (likely as a result of the small sample size), it is consistent with published data using the same ctDNA assay in another small cohort of EBC patients.^5^ Similar studies using larger non-breast cancer cohorts consistently demonstrate that ctDNA detection in either the first post-surgical sample or in any follow-up sample is associated with poorer RFS.^14^^,^^20^, ^21^, ^22^, ^23^

In addition, previous work using digital PCR (dPCR) assays showed that patients with ctDNA detected either at the first post-surgical sample or during follow-up exhibited poorer disease-free survival when compared with consistently ctDNA-undetected patients.^3^ Similarly, in a larger prospective cohort of patients with primary breast cancer (*n* = 170), those with MRD positivity during follow-up were also found to have poorer RFS, although some of these patients received neoadjuvant chemotherapy before surgery.^4^ Collectively, these results suggest that ctDNA detection at a single post-surgical timepoint may accurately predict early disease recurrence, however it is increasingly evident that longitudinal monitoring will increase sensitivity of recurrence detection particularly in the setting of prolonged risk characteristic of HR+/HER2− disease. Most of these patients remained on endocrine therapy during follow-up, which potentially could impact ctDNA detection rates. It would be important to observe the clinical course for this subset of patients after completion of endocrine therapy.

Taken together, these results suggest that detection of ctDNA post-operatively could offer a route to identify those patients with a higher likelihood of benefit from adjuvant chemotherapy, while helping to identify those patients with undetected ctDNA who might not derive additional benefit from adjuvant chemotherapy. Notably, subtype-specific therapy such as endocrine therapy and/or anti HER2+ therapy may modify the outcomes in patients with persistent ctDNA presence after the completion of adjuvant chemotherapy. Despite the limited statistical power and presence of additional subtype specific regimens received by the patients, our observations are thought provoking and require further validation in a large prospective cohort of patients. For example, recruitment to TRAK-ER (NCT04985266) is ongoing through the Royal Marsden NHS Foundation Trust, which will investigate the benefit of adjuvant therapy on patients with HR+/HER2− EBC with detectable ctDNA using an alternative method of tumor-informed ctDNA assessment.

Although there is literature regarding the utility of ctDNA detection in early-stage breast cancer as a prognostic biomarker during^24^ and after neoadjuvant therapy,^25^ the utility of ctDNA monitoring after primary surgery is yet to be validated. Our data suggest that MRD detection after primary surgery for EBC is prognostic of patient outcomes and may be modified with adjuvant therapy. Some patients relapsed, however, despite the absence of ctDNA detection in their post-operative sample. While this could be interpreted as the need for improved analytical sensitivity at a single timepoint, one must also consider the value of longitudinal ctDNA monitoring to account for the impact of underlying indolent or dormant tumor biology on ctDNA shedding. Additionally, our data suggest that combining both preoperative and post-operative assessment may further improve the ability to risk-stratify patients.

Our study possesses several limitations. We analyzed a small cohort of patients (*n* = 48) with a limited number of distant recurrences (*n* = 7). Our findings might have also been limited by the heterogeneity of the cohort employed in the study. The assay employed might lack the required sensitivity in detecting ultra-low levels of ctDNA in the immediate post-operative setting, and we were limited by the number of samples available to investigate clearance during follow-up, as well as complete data on adjuvant treatment of some patients. Given the retrospective nature of this investigation, there may have been an inherent bias in patient selection for ctDNA analysis. Despite these limitations, we propose that ctDNA analysis after primary surgery has the potential to reliably predict relapse and may facilitate adjuvant therapy decision-making in patients with EBC.

In conclusion, detection of ctDNA after surgery and before any adjuvant therapy, was associated with a high risk of distant metastasis. Patients without ctDNA detected had a very good outcome in this small series. Our data support further investigation of ctDNA after surgery and whether intervention after ctDNA detection improves outcomes, and if interventions such as endocrine therapy and/or anti-HER2 therapy may modify outcomes in patients with persistent ctDNA presence after completion of adjuvant chemotherapy.
