# Supplementary material for: Association of post-operative ctDNA detection with outcomes of patients with early breast cancers
Source: ESMO Open. 2024 Aug 30;9(9):103687. doi: 10.1016/j.esmoop.2024.103687 (PMC11402396; doi:10.1016/j.esmoop.2024.103687)
Supplement: Supplementary Figures [file mmc1.docx]

**
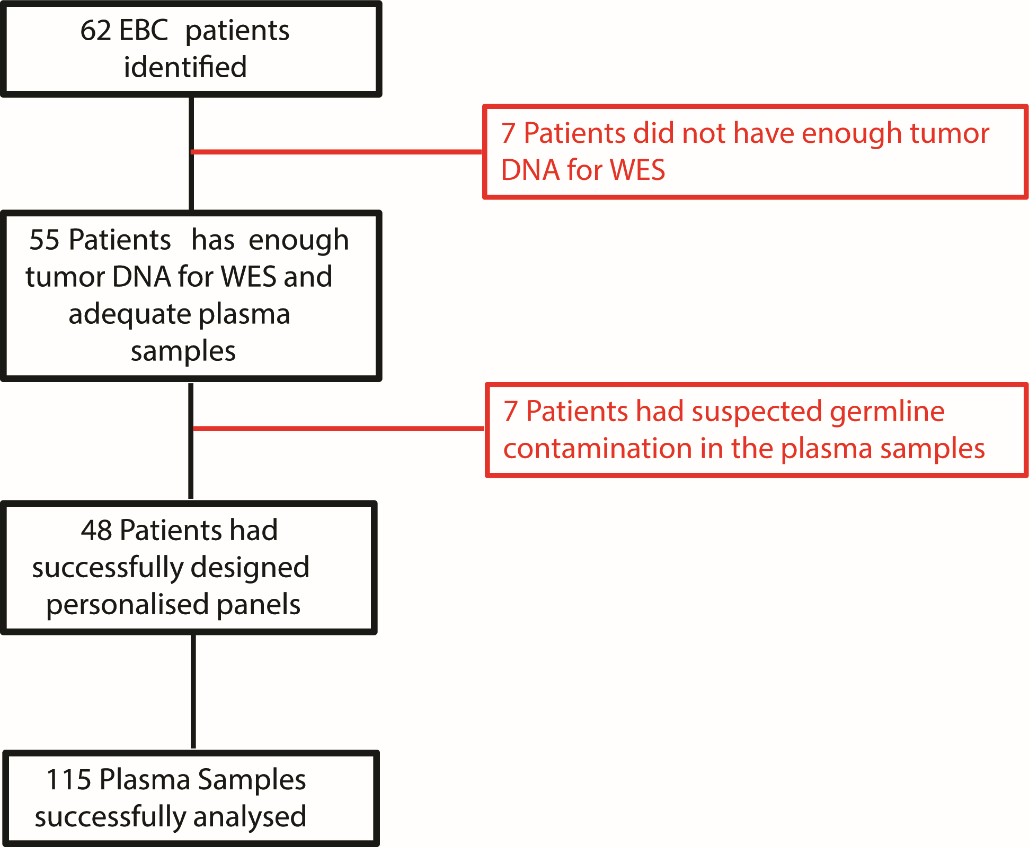
**

**Supplementary Figure 1:** CONSORT Diagram for the patients included.

**
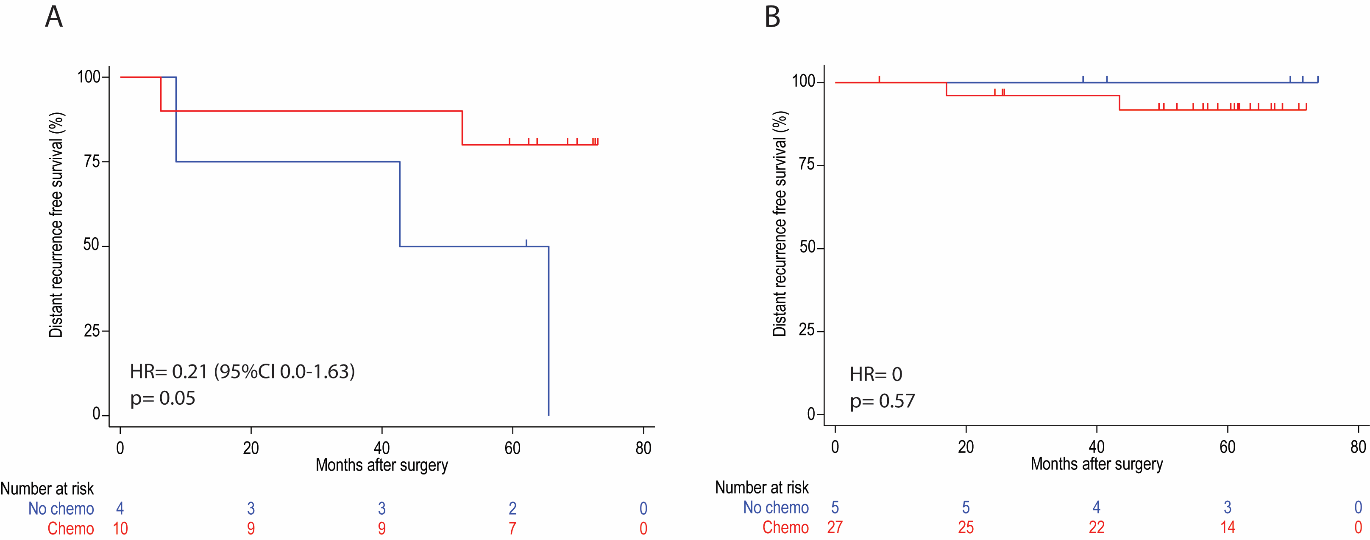
**

**Supplementary Figure 2:** **A)** Patients with ctDNA detection after surgery who did not receive adjuvant chemotherapy demonstrate a trend towards inferior relapse free survival compared to those with ctDNA detection and adjuvant chemo (log rank test). **B)** Survival of patients without ctDNA detection after surgery in the absence of adjuvant chemotherapy is not different from those who received adjuvant chemotherapy while undetectable by ctDNA after surgery (log rank test).

**Supplementary Table 1:** Clinical and histological characteristics of patients in the study.

**Supplementary Table 2:** Clinical and histopathological characteristics for all patients included in the study by receptor subtype.
